# New classification of lunate fossa fractures of the distal radius

**DOI:** 10.1186/s13018-016-0455-1

**Published:** 2016-10-21

**Authors:** Jun Zhang, Xin ran Ji, Ye Peng, Jian tao Li, Li hai Zhang, Pei fu Tang

**Affiliations:** 1 Department of Orthopaedics, Chinese People’s Liberation Army General Hospital (301 Hospital), Beijing, 100038 China; 2 Department of Orthopaedics, The Affiliated Hospital of Inner Mongolia Medical University, Hohhot, 010000 Inner Mongolia China; 3 General Hospital of PLA, Haidian District 28, Beijing, 100038 China

**Keywords:** Die-punch fracture, Radius, Classification

## Abstract

**Background:**

A die-punch fracture is a depression fracture of the lunate fossa of the distal radius. We propose a morphological classification of die-punch fractures that includes five types: center depression fractures, vertical depression fractures, volar depression fractures, dorsal depression fractures, and double die-punch fractures.

**Methods:**

The radiographs of 112 die-punch fractures treated between January 2005 and January 2015 were retrospectively reviewed. The clinical images were examined independently for two rounds by six orthopedists with different clinical experiences: two residents, two attending physicians, and two consultants. A category-specific kappa score and a kappa score for more than two observers were analyzed. We used Cohen’s kappa to test intraobserver variation.

**Results:**

The kappa score for interobserver reliability was 0.69 for the first round and 0.70 for the second round. The intraclass correlations were 0.65 and 0.63, respectively. Intraobserver reproducibility using Cohen’s kappa test was satisfactory. All of the results indicated a kappa value >0.4, suggesting good agreement within, as well as between, observers.

**Conclusions:**

The outcome was assessed using kappa statistics, which showed good interobserver reliability and intraobserver reproducibility.

## Background

Distal radius fractures are common upper limb injuries in persons of any age. Many classifications have been proposed for distal radius fractures [1]. The AO [2], Universal [3], and Fernandez [4] classifications are among those most frequently used. However, there are no detailed descriptions of the characteristics of the die-punch fracture in the existing classifications. A die-punch fracture is a depression fracture of the lunate fossa of the distal radius that is caused by a vertical load through the lunate. We believe that the previous definition of a die-punch fracture is not sufficiently comprehensive, making it easy to forget or ignore its diagnosis and treatment. In other cases, the die-punch fracture is in a state that is difficult to reset, such as when it is not limited to the lunate fossa. Therefore, redefinition of die-punch fractures can reduce the lack of attention to rare fractures, improve the accuracy of the reduction, and thus improve the treatment effect.

Five types of lunate fossa depression fracture have been found in our clinical practice, including center depression fractures, vertical depression fractures, volar depression fractures, dorsal depression fractures, and double die-punch fractures. This new classification aims to indicate the severity of the injury and thus project a prognosis based on the presumed complexity of the bone injury. Orthopedic surgeons use classification systems as a guide for treatment and prognosis as well as for comparing results in clinical studies. It is therefore of paramount importance that these systems are reliable and reproducible. All classification systems should be subjected to reliability and reproducibility testing prior to acceptance into widespread use.

The aim of this study was to establish a classification of die-punch fractures of the distal radius based on plain radiographic findings. Both intraobserver reproducibility and interobserver reliability of this classification were examined.

## Methods

### Patients

This study was designed according to previously proposed guidelines for reliability studies of fracture classification systems [5, 6]. The clinical images of 112 patients with die-punch fractures treated at our institution from January 2005 to January 2015 were retrospectively reviewed. Patients with the following conditions were excluded: distal radius fractures not involving the lunate fossa, fractures involving other than the distal radius, and patient age <18 years. No radiographs were excluded because of poor quality.

### Clinical images

Standard anteroposterior and lateral radiographs and computed tomography (CT) images of the distal radius obtained within 6 days of the injury were reviewed. The anteroposterior radiographs were obtained with the patient seated, arm abducted, elbow flexed, and hand and wrist laid flat on the radiography cassette. The wrist was in neutral position, and the vertical X-ray beam was centered on the wrist. Lateral radiographs were obtained in the true lateral position. A well-positioned lateral view shows the pisiform superior to the dorsal cortex of the distal pole of the scaphoid and the volar cortex of the capitate.

### Classification method

**Fig. 1:**
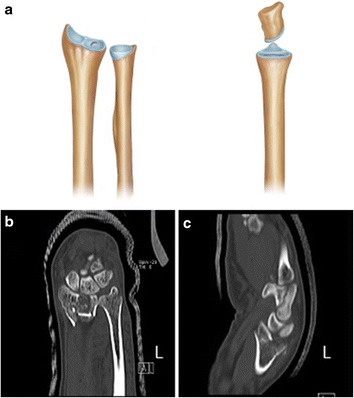
Center depression fracture. **a** The depression fracture occurs in the center of the lunate fossa of the distal radius (*left*, anterior view; *right*, lateral view). **b**, **c** Computed tomography (CT) (left)

**Fig. 2:**
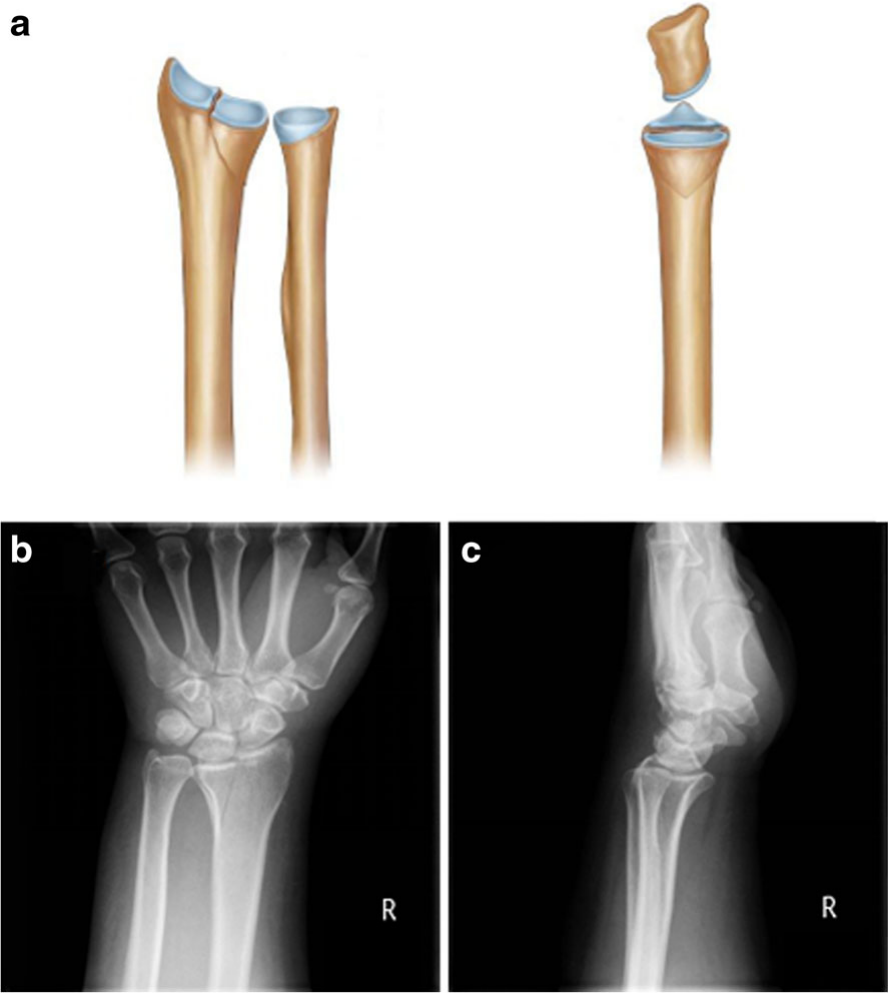
Vertical depression fracture. **a** The entire lunate fossa is vertically depressed (*left*, anterior view; *right*, lateral view). **b**, **c** Anteroposterior and lateral radiographs (right)

**Fig. 3:**
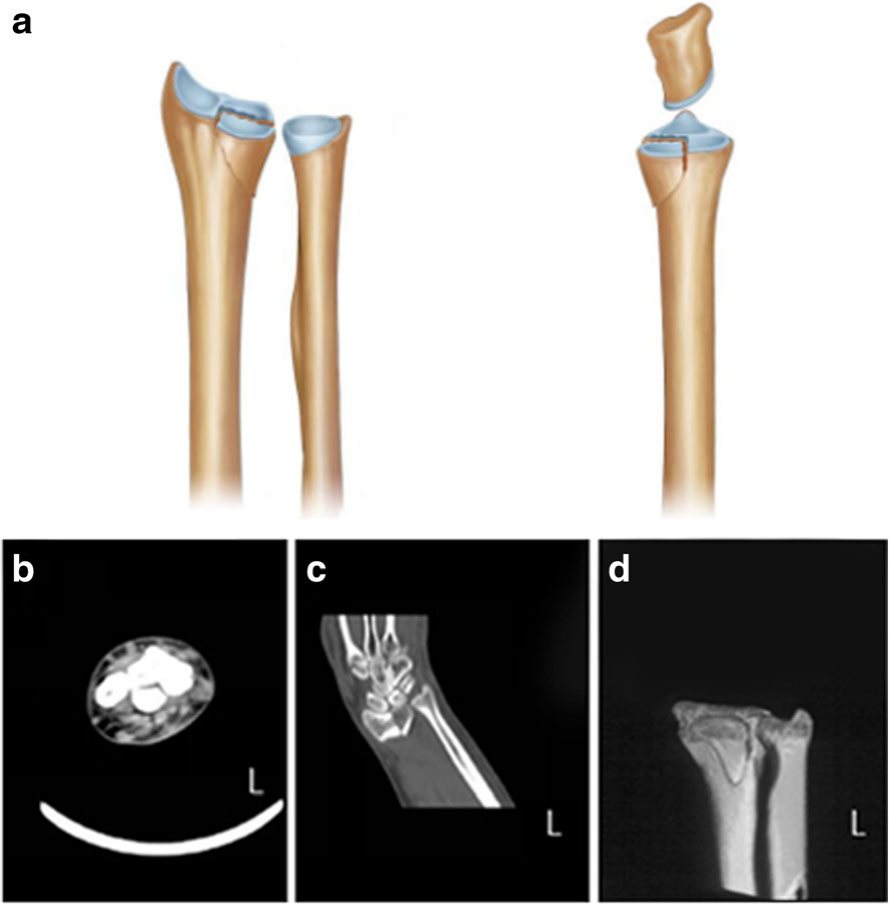
Volar depression fracture. **a** The volar half of the lunate fossa is depressed (*left*, anterior view; *right*, lateral view). **b–d** CT and three-dimensional CT (left) scans

**Fig. 4:**
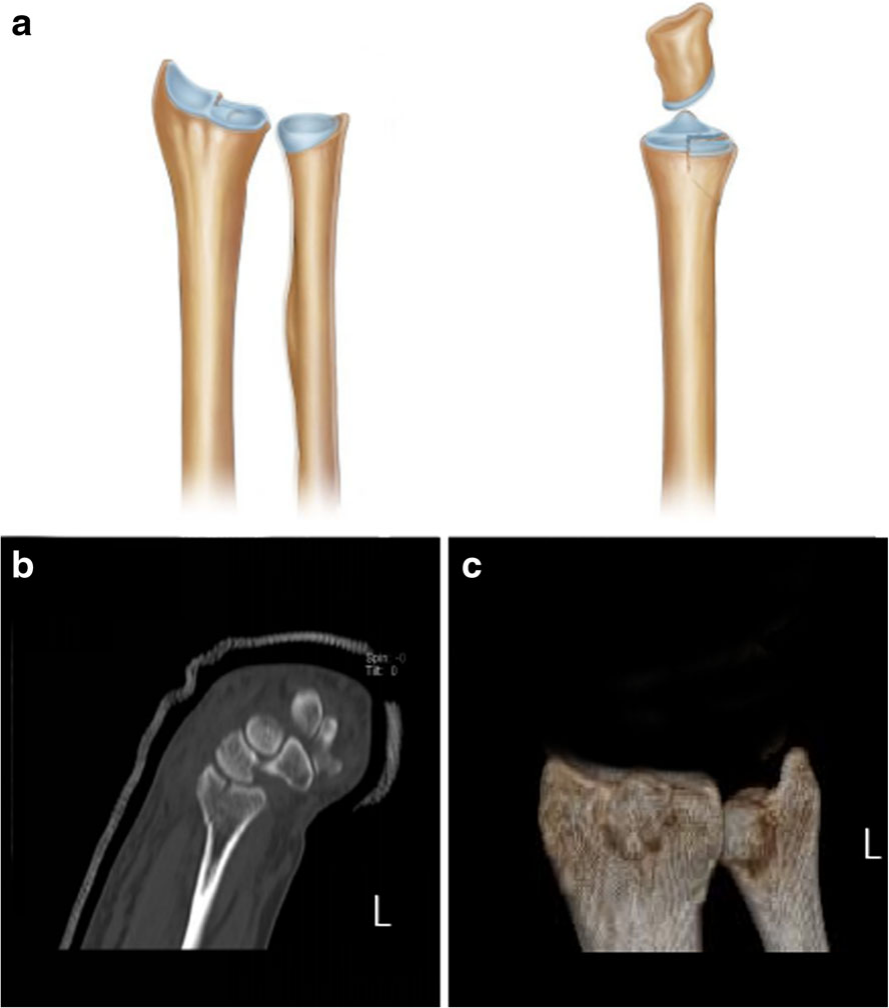
Dorsal depression fracture. **a** The dorsal half of the lunate fossa is depressed (*left*, anterior view; *right*, lateral view). **b**, **c** CT and three-dimensional CT (left) scans

**Fig. 5:**
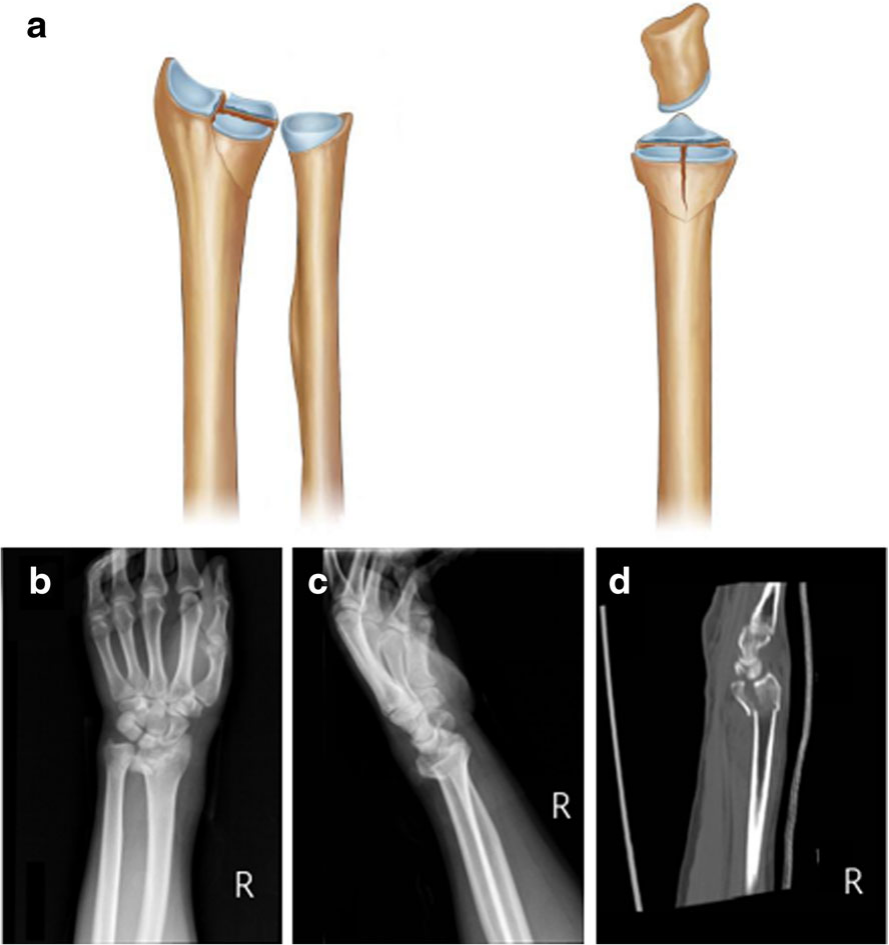
Double die-punch fracture. **a** The lunate fossa is split into two halves, each depressed toward the volar and dorsal directions, respectively (*left*, anterior view; *right*, lateral view). **b–d** Anteroposterior lateral radiograph and CT scans (right)

The clinical images were examined independently for two rounds by six orthopedists with different clinical experiences: two residents, two attending physicians, and two consultants. The die-punch fractures were classified into five types: center depression fractures (Fig. 1), vertical depression fractures (Fig. 2), volar depression fractures (Fig. 3), dorsal depression fractures (Fig. 4), and double die-punch fractures (Fig. 5).

### Statistical analysis

Statistical analyses were performed using SPSS 19 software (IBM, Armonk, NY, USA) and the associated IRR package [7]. A category-specific kappa score and a kappa score for more than two observers were analyzed [8]. We used Cohen’s kappa to test intraobserver variation [9]. A kappa score of 1 indicates perfect agreement, and a score of zero indicates that the variation in agreement can be explained purely by chance. Analysis of variance and *χ* ^2^ tests were carried out where appropriate to compare the five fracture types. A value of *P* < 0.05 was considered to indicate statistical significance.

## Results

The mean age of our patients was 59.3 years (range 18–87 years). The patient groups of different fracture classifications did not differ significantly in regard to age, sex, weight, height, body mass index, osteoporosis, or smoking status (Table 1). The percentages of patients with the various fractures were as follows: center depression fracture 14 % (15/112), vertical depression fracture 27 % (31/112), volar depression fracture 11 % (12/112), dorsal depression fracture 39 % (44/112), and double die-punch fracture 9 % (10/112).

**Table 1:** Demographics of the patients (*n* = 112)

	Center depression (*n* = 15)	Vertical depression (*n* = 31)	Volar depression (*n* = 12)	Dorsal depression (*n* = 44)	Double die-punch (*n* = 10)
Female (*n*, %)	8 (53.3)	19 (61.3)	8 (66.7)	28 (63.6)	6 (60)
Age (years)	56.2 (18 to 78)	58.9 (20 to 85)	59.1 (25 to 87)	59.6 (24 to 86)	60.1 (20 to 86)
Weight (kg)	70.6 (61 to 79)	71.1 (61 to 84)	69.9 (62 to 78)	71.4 (61 to 84)	70.5 (61 to 79)
Height (cm)	163.4 (153 to 179)	161.2 (153 to 180)	162.5 (154 to 183)	164.1 (154 to 179)	163.6 (153 to 181)
Body mass index (kg/m^2^)	26.9	27.4	28.1	27.7	27.3
Osteoporosis (%)	4 (26.7)	6 (19.4)	3 (25)	7 (15.9)	3 (30)
Smoking	2 (13.3)	2 (6.5)	3 (25)	4 (9.1)	3 (30)

The kappa score for interobserver reliability was 0.69 for the first round and 0.70 for the second round (Table 2). The intraclass correlations were 0.65 and 0.63, respectively. Intraobserver reproducibility using Cohen’s kappa test is shown in Table 3. All of the results indicated a kappa value of >0.4, suggesting good agreement within, as well as between, observers.

**Table 2:** Kappa statistics for interobserver reliability assessment

	Kappa result	Intraclass correlation coefficient
First round	0.69	0.65
Second round	0.70	0.63

**Table 3:** Kappa statistics for intraobserver reproducibility assessment

	Cohen’s kappa
Junior orthopedic resident (first round)	0.61
Junior orthopedic resident (second round)	0.59
Senior orthopedic surgeon (first round)	0.67
Senior orthopedic surgeon (second round)	0.64
Experienced orthopedic surgeon (first round)	0.69
Experienced orthopedic surgeon (second round)	0.61

## Discussion

The die-punch fracture was first described by Scheck et al. in 1962 as a dorsal fracture fragment of the lunate fossa at the distal radius [10]. The current concept of die-punch fracture is a depression fracture of the lunate fossa of the distal radius. There is no consensus on the classification or treatment guidelines for die-punch fractures. In our practice, five types of die-punch fractures have been found, including center depression fractures, vertical depression fractures, volar depression fractures, dorsal depression fractures, and double die-punch fractures. Among our patients, the dorsal depression fracture was the most frequent classification (44/112, 39 %), which might be related to the volar inclination of the distal radius articular surface. In addition, the dorsal side of the articular surface is higher than the volar side, which causes the dorsal side of the articular surface to be more prone to injury upon impact. In addition, we found that there was no statistical difference between fracture type and the patient’s sex, age, weight, height, body mass index, presence of osteoporosis, or smoking status.

In the AO classification, fractures involving the articular surfaces are classified into types B and C. Type B fractures are usually caused by light trauma and involve only the local articular surface. Although the local articular surface can be comminuted, the entire articular surface and the metaphysis are not comminuted in type B fractures. Accordingly, our study excluded patients whose fractures involved areas other than the distal radius, such as those with metaphysis of the distal radius. Therefore, in our study, the die-punch fracture was restricted to the ulnar half of the articular surface at the distal radius, which is caused by depression of the lunate fossa. In this condition, the radial half of the articular surface at the distal radius remains intact. Thus, the integrity of the distal radius is not completely destroyed. Our classification of the die-punch fracture is based on this definition of lunate fossa depression at the distal radius.

The main advantage of our classification is that observers are able to classify the fracture objectively according to computed tomography faultage parameters. They would not have to offer a judgment based on a subjective evaluation. Based on the principle of the practicality and reproducibility of a fracture categorization, we developed this classification to guide clinical treatment and infer a straightforward prognosis.

The wrist ligament is attached to the edge of the radius joint. Hence, a die-punch fracture would be difficult to reset using closed reduction. Because of the severe instability of volar and dorsal depression fractures, the displacement may reappear with the passage of the time even if the initial displacement is not severe. Moreover, according to Rikli’s three-column theory [11], about 40 % of the axial load is transferred by the lunate fossa, so anatomical reduction of the lunate fossa is very important. A non-locking steel plate should be fixed on the side where the replacement is unstable.

For a type I center depression fracture, the reset should be achieved with a small window via a volar or dorsal approach combined with bone grafting. In addition, if the collapse is >5 mm or osteoporosis is present, the joint surface should be buttressed with a steel plate. For type II vertical depression fractures, the use of a volar plate could obtain satisfactory results. For type III volar depression fractures, a modified Henry volar approach can be applied, fixing a steel plate on the palm side. A volar incision could be less affected upon tendon. For type IV dorsal depression fractures, a dorsal approach with fixing a steel plate at the back side is suggested. The multi-angle stability of a locked steel plate makes it possible to achieve fixation of the back side of the bone using a steel plate on the palm side. For type V double die-punch fractures, if fixation of a steel plate on the palm side cannot reset the bone on the back side, the dorsal approach should be applied. To obtain maximum holding force, distal screws placed under the subchondral bone can enhance the stability of the fixation, especially in osteoporotic patients. Type I and V fractures are difficult to treat surgically as they are prone to poor resolution on the joint surface, followed by emergence of traumatic arthritis of the wrist. Hence, it is essential to restore the flatness of the joint surface to the extent possible.

## Conclusions

The classification of die-punch fractures described herein is based on the injuries typically seen in our department. Therefore, it is applicable to clinical practice, which is relevant when assessing a classification. However, the reproducibility of our classification and its reliability for guiding the clinician to appropriate treatment and prognostic judgment must be further confirmed and perfected in the clinical setting.
